# A Combined Mechanochemical and Calcination Route to Mixed Cobalt Oxides for the Selective Catalytic Reduction of Nitrophenols

**DOI:** 10.3390/molecules25010089

**Published:** 2019-12-25

**Authors:** Lorianne R. Shultz, Bryan McCullough, Wesley J. Newsome, Haider Ali, Thomas E. Shaw, Kristopher O. Davis, Fernando J. Uribe-Romo, Matthieu Baudelet, Titel Jurca

**Affiliations:** 1 Department of Chemistry, University of Central Florida, 4111 Libra Drive, Orlando, FL 32816, USA; lorishultz@knights.ucf.edu (L.R.S.); mcculloughbp21@knights.ucf.edu (B.M.); wesnewsome@knights.ucf.edu (W.J.N.); tshaw@knights.ucf.edu (T.E.S.); 2 Renewable Energy and Chemical Transformations Cluster, University of Central Florida, 4353 Scorpius Street, Orlando, FL 32816, USA; 3 National Center for Forensic Science, University of Central Florida, 12354 Research Parkway #225, Orlando, FL 32826, USA; 4 Department of Materials Science and Engineering, University of Central Florida, Orlando, FL 32816, USA; alihaider@knights.ucf.edu (H.A.); Kristopher.Davis@ucf.edu (K.O.D.); 5 CREOL—The College of Optics & Photonics, Building 53, University of Central Florida, 4304 Scorpius Street, Orlando, FL 32816, USA; 6 NanoScience Technology Center, University of Central Florida, Orlando, FL 32826, USA

**Keywords:** nitroaromatics, catalytic reduction, metal oxide catalysis, waste valorization, water chemistry, nanocatalysis

## Abstract

Para-, or 4-nitrophenol, and related nitroaromatics are broadly used compounds in industrial processes and as a result are among the most common anthropogenic pollutants in aqueous industrial effluent; this requires development of practical remediation strategies. Their catalytic reduction to the less toxic and synthetically desirable aminophenols is one strategy. However, to date, the majority of work focuses on catalysts based on precisely tailored, and often noble metal-based nanoparticles. The cost of such systems hampers practical, larger scale application. We report a facile route to bulk cobalt oxide-based materials, via a combined mechanochemical and calcination approach. Vibratory ball milling of CoCl_2_(H_2_O)_6_ with KOH, and subsequent calcination afforded three cobalt oxide-based materials with different combinations of CoO(OH), Co(OH)_2_, and Co_3_O_4_ with different crystallite domains/sizes and surface areas; Co@100, Co@350 and Co@600 (*Co@###; # =* calcination temp). All three prove active for the catalytic reduction of 4-nitrophenol and related aminonitrophenols. In the case of 4-nitrophenol, Co@350 proved to be the most active catalyst, therein its retention of activity over prolonged exposure to air, moisture, and reducing environments, and applicability in flow processes is demonstrated.

## 1. Introduction

**Figure 1:**
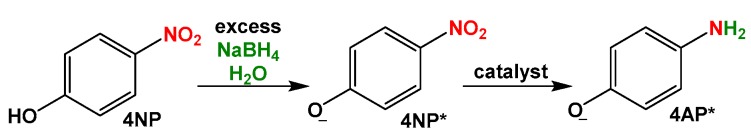
Catalytic reduction of 4-nitrophenol (4NP) to 4-aminophenolate (4AP*).

Para-, or 4-nitrophenol (4NP), and more broadly, compounds in the nitroaromatic family are widely used in the industrial fabrication of pharmaceuticals, explosives, pesticides, pigments, and dyes [1,2,3,4]. As a result, nitroaromatics have become a very common anthropogenic pollutant arising from aqueous industrial effluent. While many nitroaromatics have been shown to display acute toxicity, are mutagenic, and either potential or established carcinogens [5,6,7], their reduction products, aniline derivatives, are typically less toxic, commercially important synthetic intermediates. For example, the reduction product of 4NP is 4-aminophenol (4AP); a useful component for the synthesis of dyes, agrochemicals, and pharmaceuticals [8,9,10,11]. As a result, the aqueous catalytic reduction of 4NP has emerged as an important research area (Figure 1) [12]. Due to its concomitant use as a probe reaction system for heterogeneous catalyst development, a majority of this work, to date, has focused on noble metal nanoparticles (e.g., Au, Pt, Pd, etc.) [13,14,15]. However, although this chemistry is effectively developed with noble metal nanoparticles [16,17,18,19,20,21,22,23,24,25,26,27,28,29], their cost precludes practical scaled-up implementation. As a result, a number of methods utilizing materials that are either based on Earth-abundant metals [30,31,32,33,34,35,36,37,38] or are metal free [39,40,41,42,43,44,45,46] have emerged. Therein, cobalt oxide nanoparticles have drawn significant interest as active, low-cost alternatives to the noble metal systems [47,48,49,50,51,52]. Such species are prepared by solution-based routes commensurate with conventional nanoparticle synthesis. Although promising, the effort required for precise nanoparticle preparation can be inhibitory towards practical larger-scale applications.

Herein, we report a facile route to bulk cobalt oxide-based materials, via vibratory ball milling [53] and subsequent calcination. Vibratory ball milling, utilizing the SPEX^®^ 8000 model, as used for herein, draws ca. 249 W, comparatively, a standard hot plate stirrer draws ca. 10 W for stirring alone, and peaks at 1.5 kW to maintain a modest temperature of 80 °C, commensurate with milder hydrothermal processes for nanoparticle synthesis. By taking into consideration that mechanochemical processes are typically conducted over a shorter time span than conventional solution processes, the energy savings become additive, thus, enhancing sustainability [54]. Furthermore, the process utilizes inexpensive starting materials, no solvent during synthesis, and only deionized water (DIW) for workup. The bulk material, calcined at three different temperatures (100, 350, and 600 °C) respectively denoted as Co@100, Co@350 and Co@600, and composed of increasing amounts of Co_3_O_4_ as a function of temperature, displays different catalytic activity, and surprisingly different selectivity when utilized for the reduction of 4NP, and several aminonitrophenols. All catalytic rates observed are competitive with related literature process. The most active catalyst, Co@350 as deduced from 4NP reduction trials (vide infra) is robust, air and moisture resistant, displaying longevity, excellent recyclability, and applicability in flow processes. Paired with the ease of synthesis, and the inherent scalability of mechanochemical processes [55,56,57,58,59,60,61,62,63], these results are promising for the development of inexpensive, robust, and sustainable bulk catalysts capable of reducing nitrophenol pollutants in aqueous media.

## 2. Results and Discussion

### 2.1. Catalyst Synthesis

**Figure 2:**
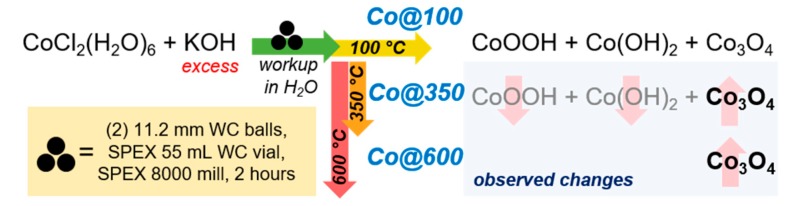
General protocol for the preparation of Co@100, Co@350, and Co@600.

In a tungsten carbide (WC) vial, CoCl_2_(H_2_O)_6_ (4.2 mmol, 1.0 g) was mixed with excess KOH (89 mmol, 5 g) and (2×) 11.2 mm WC balls. The vessel was sealed, and placed on a SPEX^®^ 8000 mill and allowed to react for 2 h after which point it was opened and the dark brown powder washed with 100 mL of DIW, passed through a medium glass filter frit to remove large particulates, and centrifuged at 3500 rpm for 3 min. to afford a dark bronze solid. The solid was dried in vacuo at 100 °C for 4 h to remove the remaining H_2_O, yielding 387 mg of dark brown/black powder. The resulting powder was split in three (129 mg each), one portion was kept “as synthesized” (Co@100) and the remaining two heated to 350 °C (110 °C h^−1^ for 3 h, hold 2 h at 350 °C) and 600 °C (110 °C h^−1^ for 5 h, hold 2 h at 600 °C), respectively (Co@350 and Co@600, Figure 2). Unless otherwise noted, samples were stored in sealed vials in a desiccator (10% RH).

### 2.2. Catalyst Characterization

**Figure 3:**
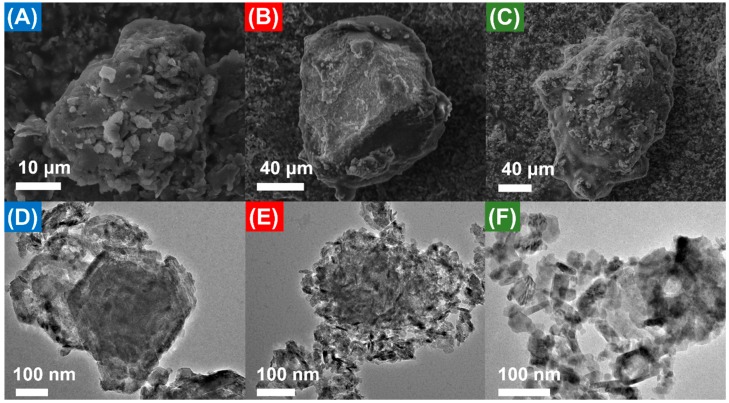
Representative SEM images of Co@100 (**A**), Co@350 (**B**), and Co@600 (**C**), and representative TEM images of Co@100 (**D**), Co@350 (**E**) and Co@600 (**F**) after sonication.

Scanning electron microscopy (SEM) of the powders in each instance revealed an amorphous agglomeration of micron-sized particles of compacted material decorated in highly dispersed nano-to-micron sized semi-spherical clusters (Figure 3A–C and Figure S1) [64,65,66]. The large agglomerates are attributed to the impacts from the preparative method. Energy dispersive X-ray spectroscopy (EDX) confirms the presence of Co as the predominant species, with residual K from the milling process (Figure S1). Transmission electron microscopy (TEM) of all three samples after prolonged sonication in methanol at 40 °C, to enable decomposition of the micron-sized agglomerates, reveals agglomerated nanoplates ranging anywhere between <10 nm to >50 nm (Figure 3D–F and Figure S2). Nanoplate definition is greatly enhanced for Co@600 (Figure 3F), commensurate with higher crystallinity and formation of a predominantly single Co-oxide species (vide infra).

**Figure 4:**
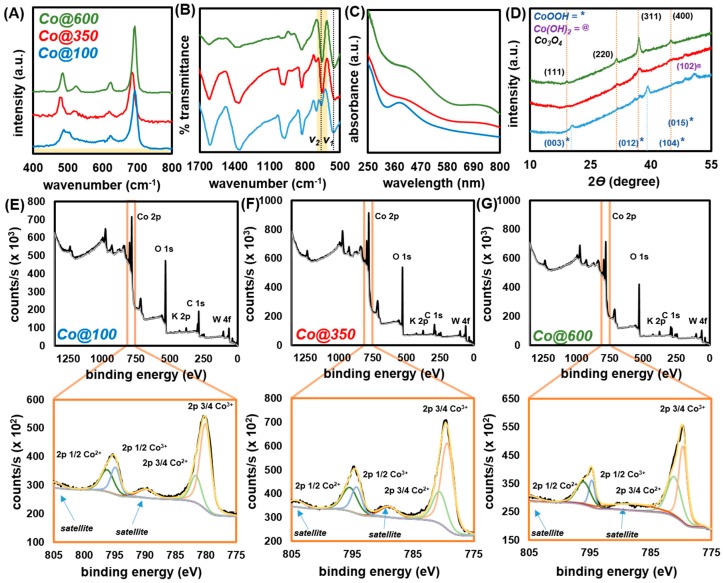
Raman (**A**), FTIR (**B**), UV-Vis (**C**), and XRD (**D**) spectra of Co@100, Co@350 and Co@600. (a.u. = arbitrary units). XPS survey and close-up of Co 2p region for (**E**) Co@100, (**F**) Co@350, and (**G**) Co@600.

Raman spectroscopy of Co@100 (Figure 4A and Figure S3) was indicative of the presence of Co_3_O_4_ with characteristic peaks at 198, 491, 528, 624, and 694 cm^−1^ corresponding to the F_2g_, E_g_, F_2g_, F_2g_, and A_1g_ modes, respectively [67,68]. However, the region of 450 to 650 cm^−1^ is sufficiently broadened and raised above the baseline. Broad peaks in this region are characteristic for CoO(OH) and Co(OH)_2_, both of which exhibit peaks at ca. 500 cm^−1^ corresponding to E_g_ and A_2u_ modes. Additionally, they both feature very broad peaks centered at ca. 600 cm^−1^ corresponding to the A_1g_ and E_g_ modes of CoO(OH) and Co(OH)_2_, respectively. The spectrum of Co@100 reveals multiple overlapping signals at both 497 and 505 cm^−1^, as well as very broad signals over the entire area of interest; likely due to the presence of both Co(OH)_2_ and CoO(OH) [67,68,69,70]. Going from Co@100 to Co@350 and Co@600, the peaks assigned to Co(OH)_2_ and CoO(OH) gradually disappear giving way to sharper features at 198, 482, 519, 621, and 693 cm^−1^ (Co@600) corresponding to the F_2g_, E_g_, F_2g_, F_2g_, and A_1g_ of Co_3_O_4_, indicating near complete conversion to the mixed oxide [67,68,71]. Additional broad features at ~130 to 300 cm^−1^ are attributed to the presence of residual KOH (Figure S3).

FTIR of Co@100 reveals a very broad high intensity peak at (*v_1_*) 551 cm^−1^ and a sharp lower intensity peak at (*v_2_*) 659 cm^−1^ (Figure 4B and Figure S2). Notably, *v_1_* is likely a combination of vibration modes for Co-O in Co_3_O_4_ spinel (Co^3+^), in CoO(OH), and in Co(OH)_2_ [72,73,74,75,76]. The sharp lower intensity *v_2_* is attributed to the vibration of Co-O in Co_3_O_4_ spinel (Co^2+^), which increases in intensity as calcination temperature increases (Co@350 and Co@600). Additional broad peaks associated with respective surface hydroxides, H_2_O (very broad at 1614 cm^−1^) [77], and carboxylates (COO^−^, at 1614 cm^−1^ and 1360 cm^−1^) [78] adsorbed and formed during handling and storage in ambient, and residual KOH from the milling process (vide supra) are also present. However, their complexity makes a clear assignment difficult. The UV-Vis spectrum of Co@100 (Figure 4C) in DIW features a broad peak at *λ*_max_ = 390 nm, characteristic of CoO(OH); but may also be attributed to the presence of β-Co(OH)_2_ [73,79]. There is significant, however ill-defined absorbance up to the as measured 800 nm region, which is attributed to the presence of Co_3_O_4_ in accordance to observations from both Raman and FTIR. Going from Co@100, to Co@350 and Co@600, the peak at *λ*_max_ = 390 nm disappears, giving way to the progressively better-defined broad bands at ca. *λ*_max_ = 430 nm and *λ*_max_ = 740 nm, attributed to the ligand-metal charge transfer events O → Co^2+^ and O → Co^3+^ of Co_3_O_4_, respectively [80].

Powder X-ray diffraction (Figure 3D) of Co@100 reveals the presence of mainly CoO(OH), with observable small amounts of Co(OH)_2_; confirmed by Raman (Figure 4D). The CoO(OH) phase appears with broad peaks (FWHM = 0.74), characteristic of the small domain size of the crystallites. Calcination to Co@350 results in phase transformation to nanocrystaline Co_3_O_4_ (spinel) with trace amounts of CoO(OH) and Co(OH)_2_. Calcination at 600 °C resulted in Co@600 with a PXRD pattern that displays sharp diffraction lines of Co_3_O_4_ (spinel) and complete disappearance of the hydroxide phases. These observations are commensurate with the spectroscopic observations. The porosity of the particles was analyzed utilizing N_2_ gas adsorption at 77 K (Figure S3). All three compounds exhibit an IUPAC type II isotherm, characteristic of microporous materials with multilayer adsorption. The hysteresis observed at high pressure accentuates the difference in the adsorption/desorption mechanism characteristic of porous oxide materials [81]. Application of the Brunauer-Emmet-Teller (BET) model over the low-pressure region (up to 0.1 *p*/*p_o_*) resulted in BET surface areas of *S*_BET_ = 21.75, 28.48, 10.38 m^2^ g^−1^ for Co@100, Co@350, and Co@600 (Figure S4).

X-ray photoelectron spectroscopy (XPS) was carried out on all three samples. As expected, measurements confirmed the majority constituents to be Co, O, and C, with a minor contaminant of K and <2% W attributed to residue from the WC grinding implements (Figure 4E–G). The presence of C is ascribed to the presence of hydrocarbon contaminants (Figure S5) [82,83]. Critical to the assessment of the material are the Co 2p peaks which reveal the ratio of Co^2+^ to Co^3+^ in the system. The observed peaks at respective binding energies (eV) and correlated % Co^2+/3+^ compositions are as follows: Co@100 2*p*_1/2_ (Co^2+^) 796.6 eV, 2*p*_1/2_(Co^3+^) 795.4 eV, 2*p*_3/4_ (Co^2+^) 781.6 eV, 2*p*_3/4_ (Co^3+^) 780.1 eV, Co^2+^ = 23.9%, and Co^3+^ = 76.1%; Co@350 2*p*_1/2_ (Co^2+^) 795.5 eV, 2*p*_1/2_(Co^3+^) 794.2 eV, 2*p*_3/4_ (Co^2+^) 780.6 eV, 2*p*_3/4_ (Co^3+^) 779.4 eV, Co^2+^ = 30.0%, and Co^3+^ = 70.0%; Co@600 2*p*_1/2_ (Co^2+^) 796.6 eV, 2*p*_1/2_(Co^3+^) 794.8 eV, 2*p*_3/4_ (Co^2+^) 781.2 eV, 2*p*_3/4_ (Co^3+^) 779.7 eV, Co^2+^ = 47.7%, and Co^3+^ = 52.3%. Thus, the compositions support the transition from predominantly CoO(OH) (Co^3+^) to predominantly Co_3_O_4_ (2:1 Co^3+^:Co^2+^) [64,84,85]. The “overshoot” in Co^2+^ content for Co@600 is potentially due to a combination of surface –OH sites or oxygen vacancies arising from the preparative process and handling under high humidity ambient conditions.

Combining the spectroscopic, diffraction, and porosity measurements, thermal treatment of cobalt-based nanoparticles first induces a structural rearrangement from mainly cobalt oxyhydroxide to a mixed system featuring both oxyhydroxide and spinel with small crystallite size and increased accessible surface area. Annealing at 600 °C results in increased particle size with a decrease in surface area with formation of predominantly Co_3_O_4_ spinel, with likely oxygen vacancies or amorphous surface hydroxides. The change in chemical environment is echoed by the initial decreased (red) shift (Co@100 to Co@350) then increased (blue) shift (Co@350 to Co@600) observed by Raman (Figure 4A).

### 2.3. Catalysis

#### 2.3.1. Catalytic Reduction of 4-Nitrophenol

**Figure 5:**
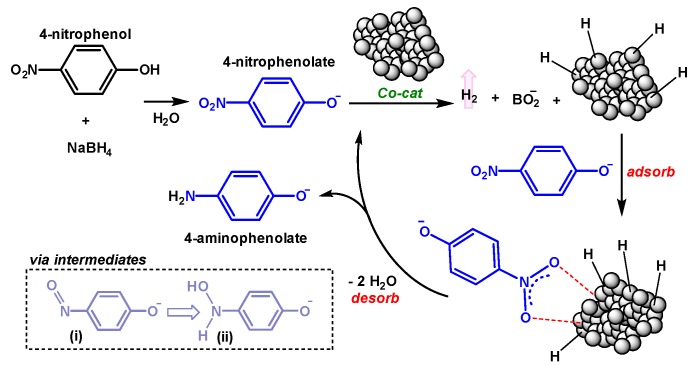
Proposed general mechanism for the reduction of 4NP with catalysts Co@100, Co@350, and Co@600 in H_2_O with excess NaBH_4_.

**Figure 6:**
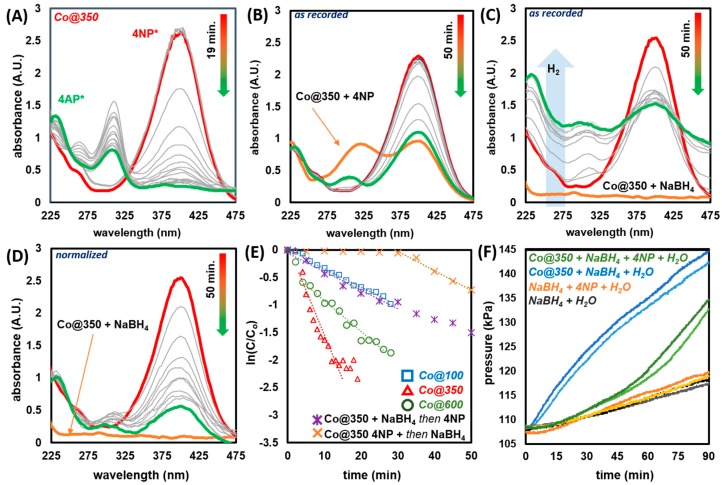
(**A**) UV-Vis spectra for the reduction of 4NP by Co_350_; (**B**) Co@350 and 4NP, dwell 5 min, then, NaBH_4_ addition; (**C**) Co@350 and NaBH_4_, dwell 5 min, then 4NP addition; (**D**) spectrum (**C**) normalized at 250 nm; (**E**) plot of ln(C/Co) as a function of time; and (**F**) change in pressure associated with the evolution of H_2_ in closed system (repeat trials shown).

To investigate the catalytic performance of Co@100, Co@350 and Co@600, 4-nitrophenol (4NP) reduction reactions were conducted in a standard 1 cm path length quartz UV cell and monitored in the 225 to 475 nm region, under ambient (21 °C). In a typical reaction, 0.39 μmol 4NP was diluted in 1 mL DIW mixed with a 2 mL solution of 0.2 mmol NaBH_4_ (pH 10.46); this initial reaction generates 4-nitrophenolate (4NP*) marked by a color change from pale to bright yellow, and a red shift from *λ*_max_ = 317 nm to *λ*_max_ = 400 nm (Figure 5, Figure 6 and Figure S6). After the *t_0_* absorbance was recorded, 1 mg of catalyst was added and stirred for ~2 s. The reaction was monitored at fixed intervals by the decreasing absorbance at *λ*_max_ = 400 nm. This was accompanied by the appearance of a lower intensity peak at *λ*_max_ = 310 nm, consistent with 4-aminophenolate (4AP*); 4-aminophenol (4AP) appears at ca. *λ*_max_ = 300 nm (Figure S6). Practically, this denotes a color change from bright yellow to colorless. Notably, all three catalysts promote hydrolysis of NaBH_4_ resulting in a turbid solution and an artificially increased baseline absorbance (~1 a.u.). The catalytic hydrolysis of NaBH_4_ by Co_3_O_4_-based species has been previously reported, and therefore it is not surprising that a similar competing reaction is operating herein [86,87,88]. To effectively extract data under these conditions, the resulting spectra were normalized to an isosbestic point at 250 nm.

When conducted in the presence of excess NaBH_4_, the reaction is commonly described in terms of the Langmuir-Hinshelwood model [13,89,90,91]. According to the model, reactants 4NP* and NaBH_4_ derived surface hydrogen are first adsorbed onto the catalyst surface in a fast and reversible process. Following an induction period (vide infra), the reaction proceeds according to pseudo-first order kinetics, via intermediates 4-nitrosophenol and 4-hydroxylaminophenol (Figure 5i,ii) [92,93,94] ultimately yielding the product 4AP*, which rapidly desorbs from the surface, thus bearing minimal impact on the overall kinetics (Figure 5). As a result, the rate-limiting step can be described as the reaction of adsorbed 4NP and hydrogen on the catalyst surface. Thus, the resulting catalyst performance is quantified in terms of the apparent rate constant *k_app_* (Equation), derived from the slope of ln(*C/C_0_*) as a function of time; *C*/*C_0_* is obtained from the absorbance for 4NP* at *λ*_max_ = 400 nm (*A*/*A_0_*), collected after an induction period (vide infra). The details governing this approximation have been discussed in prior literature [5].

$$-k_{app}t\;=\;ln\left(\frac{A}{A_{0}}\right)\;=\;ln\left(\frac{C}{C_{0}}\right)$$

Reactions promoted by all three catalysts, monitored at 2 min intervals, proceeded as expected (Figure 6A and Figure S5). Reactivity, as delineated by *k_app_* was found to proceed in the order Co@350 (0.189 min^−1^) > Co@600 (0.057 min^−1^) > Co@100 (0.033 min^−1^) (Figure 6F, Table 1). By comparison, repeating the reaction with commercial Co_3_O_4_ led to no observed reaction over the 50 min period tested (Figure S8A). Because all three catalysts feature a different composition of materials, a comparative analysis of structure-activity relationships is not possible. Within the context of Co@600, enhanced activity over pristine commercially sourced Co_3_O_4_ can be attributed to the presence of oxygen vacancies. Chen and coworkers have previously reported oxygen vacancy enhanced activity of Co_3_O_4_ towards 4NP reduction, wherein pristine Co_3_O_4_ exhibited long induction periods and low initial activity, whereas reduced species, denoted by higher Co^2+^ content by XPS, were more active [50]. Co@600 exhibits a Co composition of Co^2+^:Co^3+^ 47.7%:52.3% as compared with Co_3_O_4_ (1:2 Co^2+^:Co^3+^). The exact blend of CoO(OH), Co_3_O_4_, surface Co(OH)_2_, and relative oxygen vacancies of Co@100 and Co@350 are difficult to assign, thus their activity can more broadly be attributed to the process-specific mixture of the aforementioned components. Therein, the processing parameters leading to Co@350 yield the most active mixed cobalt oxide blend for 4NP reduction. However, Co@600 exhibits larger crystallite/domain size and decreased BET surface area (Co@350 = 28.28 m^2^ g^−1^ and Co@600 = 10.38 m^2^ g^−1^), which may account for the increased *k_app_* of Co@350.

A direct comparison to literature catalysts is complicated by the dependence of *k_app_* on multiple factors including NaBH_4_ concentration, mass transport, reaction volume, catalyst weight, temperature, and the nature and density of active sites, which in most cases, ours included, remain enigmatic. Nonetheless, it is accepted that *k_app_* normalized to reaction volume and catalyst mass, dubbed “activity parameter” *κ* (s^−1^ g^−1^ L) can facilitate a useful comparison [95,96]. Using these parameters, Co@350 exhibits a *κ* value of 9.5 × 10^−3^ s^−1^ g^−1^ L. This is comparable to a number of recent literature reports for both metal oxides; CuO (6.2 × 10^−3^ s^−1^ g^−1^ L), [7c] Co_3_O_4_ (4.2 × 10^−3^ s^−1^ g^−1^ L) [32], and meso-Co_3_O_4_ (3.8 × 10^−3^ s^−1^ g^−1^ L) [48]; metal nanoparticles Ni (2.9 × 10^−3^ s^−1^ g^−1^ L) [97], Ni on carbon (27.6 − 69.6 × 10^−3^ s^−1^ g^−1^ L) [98], and Au on Fe_3_O_4_ (61.0 × 10^−3^ s^−1^ g^−1^ L) [99]; and metal free catalysts such as sulfurized graphene (6.2 × 10^−3^ s^−1^ g^−1^ L) [46].

To confirm adherence to the Langmuir-Hinshelwood model [13,89,90,91], two additional experiments were conducted with Co@350 utilizing similar parameters as detailed prior, while altering the order of reagent addition. First, Co@350 and 4NP in DIW were introduced to the quartz cell and absorbance was recorded (Figure 6B). Notably some 4NP is immediately converted to 4NP* (normally a slower process); this is observed for all three catalysts (Figure S7) and is attributed to the presence of residual KOH. After the initial measurement, NaBH_4_ is added to the system and absorbance is subsequently measured at 5 min intervals. With this sequence of addition, there is a considerable induction time (30 min) during which negligible H_2_ evolution is observed; this is evident by the lack of turbidity of the solution which reveals no increase in baseline absorbance in the recorded spectrum (Figure 6B). The process was repeated with initial introduction of Co@350 and NaBH_4_ in DIW, measurement of absorbance and subsequent addition of 4NP with monitoring at 5 min intervals. In this instance, H_2_ evolution was immediately noticeable, and reflected in the recorded spectra which featured a dramatically increased baseline absorbance (Figure 6C, normalized values see Figure 6D). Notably, there was no observed induction time at 5 min. The reactions proceeded with a *k_app_* of 0.035 min^−1^ (Co@350 + 4NP then NaBH_4_) and 0.032 min^−1^ (Co@350 + NaBH_4_, then 4NP), indicating that the reduction process is likely similar post induction period. Ultimately, it appears the optimal scenario is the introduction of catalyst to a mixture of NaBH_4_ and preformed 4NP*, which in the case of Co@350 resulted in a *k_app_* of 0.189 min^−1^ with minimal induction time (2 min).

4NP has been shown to have a dramatically higher adsorption constant than BH_4_^−^ for Co_3_O_4_ (*K_4NP_* = 922 ± 40 L mol^−1^ and *K_BH4_-* = 22 ± 4 L mol^−1^) [48]. This is consistent with our observed induction period (30 min), where initial exposure to 4NP likely results in complete coverage of catalyst surface sites, thereby preventing co-adsorbance of BH_4_^−^ necessary to generate the surface hydrogens (which readily evolve H_2_) required for catalytic turnover. Alternatively, initial formation of surface hydrogen species does not fully out-compete 4NP adsorption even if addition is delayed, and the reaction proceeds without significant induction time, and with similar rate. This is consistent with the Langmuir-Hinshelwood model which necessitates adsorption of both species onto the catalyst surface prior to reaction [13,89,90,91]. These findings can also rule out the competing Eley-Rideal mechanism [100,101,102], which is predicated on the collision of substrate with surface-bound hydrogens; in such a case we would expect our second scenario (Co@350 + NaBH_4_ then 4NP), featuring a hydrogen rich surface and all 4NP initially in the solution state to have exhibited an enhanced *k_app_*.

In a separate experiment, the change in pressure associated with the hydrolysis of NaBH_4_ as promoted by Co@350 both in the absence and in competition with 4NP was monitored in a closed system (Figure 6F and Figure S9). In the absence of catalyst, the pressure of the system changes by Δ9.27 kPa over 90 min. due to slow hydrolysis of NaBH_4_ in H_2_O. Introduction of Co@350 results in enhanced H_2_ production, resulting in an additional pressure change of Δ25.59 kPa. Overall this represents a nearly four-fold increase in hydrolysis rate, as expected for Co-based catalysts [87,88,89]. Monitoring the pressure change for the reduction of 4NP in the system reveals only slight deviation over the first ~50 min (4NP reduction period). After this point, pressure begins to increase steadily with a similar slope as observed for trials in the presence of Co@350, and the absence of 4NP. These observations are consistent with the higher adsorption constant for 4NP vs. BH_4_^−^ with Co_3_O_4_ [48] and further validate the mechanistic studies outlined in Figure 6B–D (vide supra) [13,89,90,91]. Moreover, commercial Co_3_O_4_ readily promotes NaBH_4_ hydrolysis [88], although it fails to promote 4NP reduction during the time tested (Figure S8B), thus validating that (a) it is not fully passivated and capable of being catalytically active, and (b) that the preparative routes undertaken for our materials yield cobalt oxides that are comparatively highly active towards 4NP reduction.

The presence of induction periods, as observed herein, is common in heterogeneous catalysis, and in the context of 4NP reduction, has been attributed to a number of factors. The prevailing theories center around reconstruction of catalyst surface to yield active sites [103,104,105,106], and the time for depletion of dissolved oxygen which competes with 4NP for BH_4_^−^ [107,108,109], or as proposed more recently, could promote a rapid reversal by reacting with 4AP to regenerate 4NP [110]. However, these theories arose largely from studies of well-defined noble-metal nanoparticles, as such direct appropriation to the highly amorphous metal oxide system presented herein is not straightforward, nor is a mechanistic study of the exact surface chemistry governing the observed induction period. Nonetheless, it is evident from the structural characterization that the catalyst surfaces are coated with a number of adsorbed hydroxides and carboxyl groups (vide supra) which likely necessitates an induction period to expose or form active sites. Monitoring the pH of the reduction process at 5 min intervals reveals an initial dip upon catalyst addition (Figure S10), which could be due to the release of adsorbed species and is commensurate with the induction period timescale. Overall, pH increases as the reaction proceeds, likely due to the formation of BO_2_^−^.

To delineate the effects of trace KOH, a series of experiments were conducted under similar conditions. Attempted reduction of 4NP with 1 mg of KOH in lieu of Co catalyst, and in the presence of NaBH_4_ with monitoring at 2 min intervals for 28 min resulted in no observable decrease in 4NP* absorbance, and no formation of 4AP* (Figure S11A). Repeating the process in the absence of NaBH_4_ resulted in 4NP* formation, and no further observable reaction over 28 min (Figure S11B). These findings are consistent with a prior report on nitrobenzene reduction [111]. Repeating the catalytic reduction of 4NP with Co@350 and the addition of 1 mg KOH facilitated reduction to 4AP* with a *k_app_* of 0.132 min^−1^ in 22 min (Figure S11C). This is only a slightly reduced rate as compared with the system without additional KOH (*k_app_* = 0.189 min^−1^). Yan, Xie, and coworkers have recently proposed an alternative pathway for 4NP to 4AP reduction via direct hydrogenation of 4NP to yield 4-nitrosophenol (protonated intermediate i, Figure 5), which ultimately yields 4AP/4AP*. The presence of this species accounts for an initially enhanced absorbance at *λ*_max_ = 310 nm [93]. We observe a similar enhanced absorbance at 310 nm which decreases as 4NP is consumed (Figure 6B and Figure S7). Thus, we postulate that increased concentration of K^+^ in the system, studied herein, could promote a complementary reaction pathway via 4-nitrosophenol, thus, accounting for the increased absorbance, and slight reduction in *k_app_* (δ − 0.057 min^−1^) [112,113,114,115]. Overall, it is evident that trace KOH is not the driving factor in the catalytic activity reported herein but could enhance a complementary or concurrent reaction pathway, ultimately leading to the same product.

#### 2.3.2. Recyclability of Co@350

**Figure 7:**
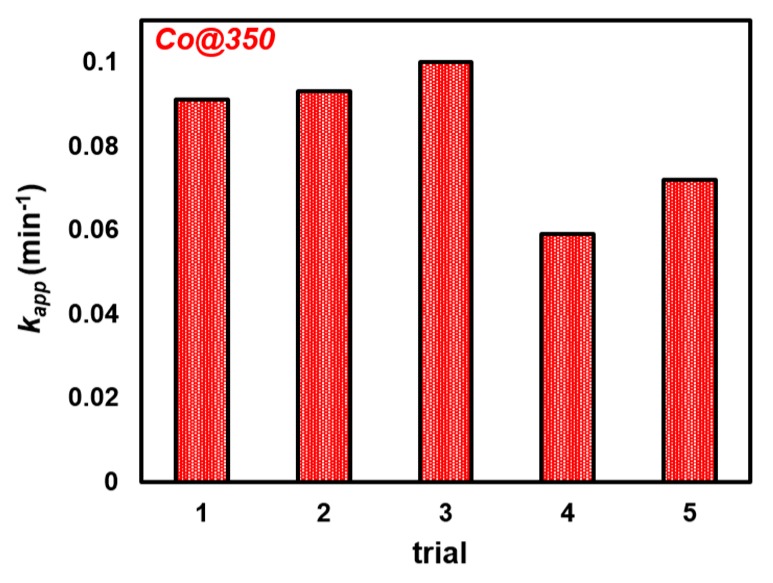
Recyclability of Co@350 for the reduction of 4NP* to 4AP.

Under identical conditions to our prior in situ catalysis trials with 4NP (vide supra), we tested the recyclability and long-term stability of Co@350; our most active catalyst. Prior to use, to ensure consistency throughout the recyclability trials, Co@350 was initially exposed to ambient conditions (45% to 55% RH). An immediate impact on performance, lowering the *k_app_* from 0.189 min^−1^ to 0.091 min^−1^ (Table 1) was observed. This is likely due to a combination of adsorbed H_2_O and CO_2_, which has the dual effect of blocking sites, and lowering the catalyst weight % of the 1 mg added to the reaction mixture. Nonetheless, Co@350 remained active under medium humidity, “real-world” conditions. After every trial, the turbid solution was allowed to settle overnight, the liquid decanted, the solid washed with DIW, again decanted, and the remaining catalyst placed under vacuum overnight. The subsequent trials were conducted by the addition of 3 mL of DIW containing 4NP and NaBH_4_, with monitoring at 5 min intervals. Over five trials, spanning nine days, there was an observed decrease in *k_app_* of 21% (Figure 7 and Figure S12). This may be due in part to degradation of catalyst; however mechanical loss during workup is likely a significant contributor. The fluctuation in *k_app_* from trial to trial is attributed to agglomeration and dispersion of particulates incurred during the workup process. Raman spectroscopy of the “spent” catalyst was indicative of predominantly Co_3_O_4_ with characteristic peaks at 184, 460, 500, 600, and 663 cm^−1^ corresponding to the F_2g_, E_g_, F_2g_, F_2g_, and A_1g_ modes, respectively (Figure S13) [67,68]. However, broadening of the peaks in the 450 to 500 cm^−1^ region, and the enhanced intensity of the signal at 460 cm^−1^ also points to the expected presence of Co(OH)_2_ and CoO(OH) [67,68,69,70]. XRD studies of spent Co@350 as well as Co@100 and Co@600 (Figure S14) indicate the expected presence of CoO(OH) and Co_3_O_4_ for Co@350 and Co@100, and Co_3_O_4_ for Co@600.

#### 2.3.3. Testing Applicability of Co@350 in a Flow Process

**Figure 8:**
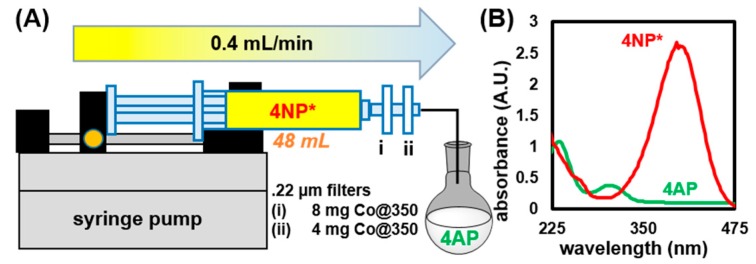
(**A**) Schematic illustration of a model flow reactor for reduction of 4NP* to 4AP. (**B**) UV-Vis spectrum of 4NP* and the resulting 4AP collected after passage through filters loaded with Co@350.

To investigate applicability of our catalyst in a flow process [116,117,118,119,120], we simulated a flow reactor with a simple proof-of-concept model. Catalyst Co@350 was loaded onto two 0.22 μm nylon syringe filters; 8 mg in filter (i) and 4 mg in filter (ii), then stacked sequentially (Figure 8A). The filters were then flushed with 10 mL of DIW to remove any loose particulates or readily dissolved species. A 60 mL syringe was loaded with 6.21 μmol 4NP, and 3.2 mmol NaBH_4_ in 48 mL of DIW. The syringe and filters were assembled and placed on a syringe pump, as illustrated in Figure 8A, where the solution was displaced at a rate of 0.4 mL min^−1^ and the product was collected in an open flask. The bright yellow solution of 4NP* became completely clear after passage through the filter, as confirmed by UV-Vis (Figure 8B).

#### 2.3.4. Catalytic Reduction of Amino-Nitrophenols

**Figure 9:**
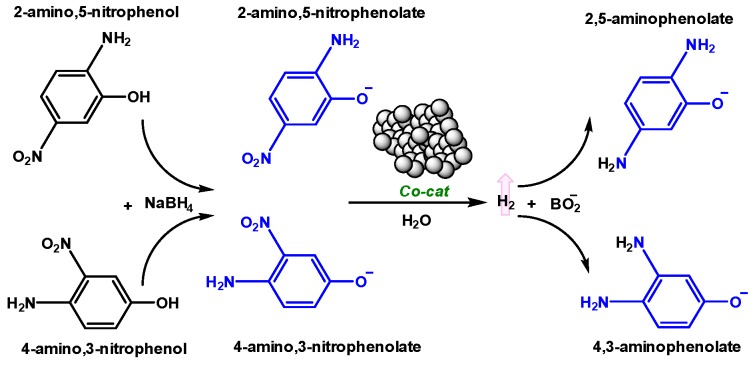
Reaction process of amino-nitrophenols with catalysts Co@100, Co@350 and Co@600 (“Co-cat”) in H_2_O with excess NaBH_4_.

**Figure 10:**
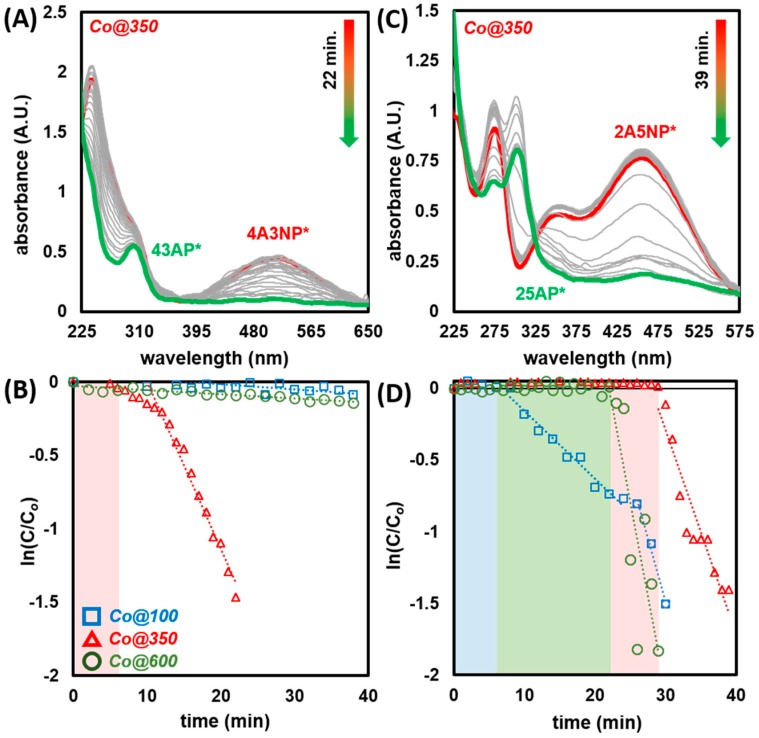
(**A**) UV-Vis spectra of the reduction of 4A3NP by Co@350 (SI for Co@100 and Co@600) and (**B**) plot of ln(C/Co) as a function of time for the reduction of 4A3NP by Co@100, Co@350, and Co@600. (**C**) UV-Vis spectra of the reduction of 2A5NP by Co@350 (SI for Co@100 and Co@600) and (**D**) plot of ln(C/Co) as a function of time for the reduction of 2A5NP by Co@100, Co@350, and Co@600. Note, color blocks represent induction times.

Encouraged by the results from the reduction of 4NP, we expanded the substrate scope to 4-amino,3-nitrophenol (4A3NP) and 2-amino,5-nitrophenol (2A5NP) (Figure 9). Therein, reactions were conducted under a similar protocol as described for 4NP. In both instances, introduction of amino-nitrophenols to a solution of NaBH_4_ in DIW led to a shift in absorbance commensurate with the formation of respective amino-nitrophenolates: 4-amino,3-nitrophenolate (4A3NP*) and 2-amino,5-nitrophenolate (2A5NP*) (Figure S4). Reactions were monitored by the disappearance of peaks at *λ*_max_ = 510 nm (4A3NP*) and *λ*_max_ = 460 nm (2A5NP*) (Figure 10, Figures S15 and S16). The recorded absorbance of the resulting diaminophenolates, 4,3-aminophenolate (4A3NP*) and 2,5-aminophenolate (25AP*), were consistent with prior literature [121,122,123,124].

In the case of 4A3NP, the results from the initial 4NP trials were accurately predictive. Within the tested time of 40 min, Co@350 rapidly facilitated reduction (*k_app_* = 0.114 min^−1^), whereas both Co@100 and Co@600 displayed only negligible activity (*k_app_* = 0.004 and 0.003 min^−1^ respectively). By switching the position of the amine and nitro groups to 2A5NP, activity across all three catalysts changed dramatically (Figure 10 and Table 1). Co@100 appeared to exhibit two distinctly different rates; after an induction time of 8 min, reduction proceeded at a modest rate (*k_app_* = 0.047 min^−1^), this increased sharply at ca. 26 min to 0.175 min^−1^. We tentatively attribute this to the multi-component nature of Co@100, wherein initial activity may be due to Co(OH)_2_ species, and the increased, latent rate to CoO(OH) or Co_3_O_4_ as available in the other catalysts. This is commensurate with both the rates and induction times observed for Co@350 and Co@600 (vide supra). The highest rate observed was for Co@600 (*k_app_* = 0.264 min^−1^) with an induction time of 22 min. The slowest catalyst was Co@350, exhibiting both the lowest rate (*k_app_* = 0.143 min^−1^) and longest induction (28 min). In this case, the observed rates for Co@350 and Co@600 inversely correlate to BET surface area (Co@350 = 28.28 m^2^ g^−1^, Co@600 = 10.38 m^2^ g^−1^). The difference in reactivity is, therefore, more likely related to the nature of available surface sites as dictated by the particle size, degree of crystallinity (vide supra), and the combination of CoO(OH) and Co_3_O_4_ available. These findings highlight the potential of fine-tuning activity and selectivity for cobalt oxide- and hydroxide-based catalysts through combined mechanochemical synthesis and thermal treatment. Furthermore, they call in to question the common practice of comparing the activity of heterogeneous catalysts based on 4NP screening alone. As noted herein, addition and respective position of functional groups to the 4NP scaffold can easily reverse observed rates from the ubiquitous 4NP model reaction [125].

## 3. Materials and Methods

All reactions were carried out under ambient (21 to 23 °C, 45% to 55% relative humidity). Cobalt(II) chloride hexahydrate, 4-nitrophenol (4NP), 4-amino,3-nitrophenol (4A3NP), 2-amino,5-nitrophenol (2A5NP), anilines, and NaBH_4_ were purchased from TCI America, KOH was purchased from Strem Chemicals, and used as received. Co_3_O_4_ (99.7%) was purchased from Alfa Aesar and used as received. FTIR Spectra were measured on a Bruker Vertex-70 with Helios ATR attachment. UV-Vis Spectra were collected on an Agilent Cary 60 spectrophotometer utilizing 1 cm quartz cuvettes. Optical microscope images were collected on a Leica DM2500m with an Accu-Scope Excelis-HD camera. The pH measurements were conducted with an OHaus 2100 m with ST210 electrode. Pressure change due to H_2_ evolution was measured in a closed system with a Vernier PS400-BTA sensor. The N_2_ gas adsorption isotherm analysis was performed using a Micromeritics ASAP 2020 surface area and porosity analyzer. Measurements were performed at 77 K (liquid N_2_ bath) on thermally activated samples. The BET surface areas were obtained by performing a Rouquerol analysis over the linear isotherm to determine the upper limits of the BET model. Least squares linear fitting over the BET plot provided the parameter for the volume of the monolayer, surface area, and C-constant, following the recommendation by Rouquerol [80]. The powder X-ray diffraction measurements were performed using a Rigaku Miniflex 600 diffractometer, with 2θ Bragg-Brentano geometry, a 300 mm goniometer diameter, and a 600 W (40 kV, 15 mA) X-ray tube source using Ni-filtered CuK_α_ (*λ* = 1.5418 Å) radiation, equipped with a high-resolution D/tex 250 detector, 5.0° incident and receiving Soller slits, a 0.625° divergent slit, a 1.25° scattering slit, a 0.3 mm receiving slit, a Ni-CuK_β_ filter, and an anti-scattering blade. Samples were measured from 10 to 55 2-theta degrees with a step size of 0.02 degrees and a scan rate of 0.5 min per step. Powder samples were prepared by depositing the powder sample on a silicon zero-background sample holder and gently pressing the powder with a glass slide. Raman data were acquired using a Horiba XploRA-plus microscope with a 785 nm laser excitation source. The laser was focused onto the samples using 100× objective producing a spot size of ~1 μm. Spectra were acquired from 20 to 2000 cm^−1^ with 10 s acquisition time. The SEM images were acquired with a LEO 1450VP and accompanied by an Oxford EDX for elemental analysis. All samples were mounted on metal and silver-coated to provide enhanced contrast images. Bright field (BF) images were obtained with the help FEI Tecnai F 30 TEM in conventional transmission electron microscopy (CTEM) mode at an operating voltage of 300 kV. The X-ray photoelectron spectra (XPS) were obtained using a Thermo Scientific ESCALAB XI+ X-ray Photoelectron Spectrometer with an Al Kα X-ray source (1486.67 eV). The TEM samples were prepared by prolonged sonication of samples in methanol at 40 °C followed by dispersion on CF-2/4-3Cu-25 C-flat^TM^ holey carbon on copper grids from Electron Microscopy Sciences.

## 4. Conclusions

Reduction of nitrophenols is typically carried out by either noble-metal nanoparticles, or alternatives synthesized with precise control over size and shape. This requires a significant expenditure of either material cost, experimental effort, or in many cases, both. These factors can present an obstacle to any practical, large-scale application as would be necessitated for environmental remediation. The process reported, herein, represents a facile route to bulk cobalt-oxide based materials, via a combined mechanochemical (vibratory ball milling) and calcination approach. By utilizing inexpensive starting materials such as CoCl_2_(H_2_O)_6_ and KOH, no solvent during synthesis, and only DIW for workup, it remains both economical and sustainable. Calcination at 100, 350, and 600 °C afforded three materials (Co@100, Co@350 and Co@600) composed of increasing amounts of Co_3_O_4_ and decreasing amounts of CoO(OH). All materials can generally be described as micron-sized compacted agglomerates of nanoplates. All three were active for the reduction of 4NP, with activity varying based on a combination of chemical composition, crystallinity, and surface area. Therein, Co@350 displayed the best performance and was further studied for recyclability and applicability in a flow process, where it was shown to remain active after prolonged exposure to air and moisture.

Expanding the substrate scope to amine-functionalized nitrophenols revealed unexpected results. While 4A3NP reflected the trends observed with the 4NP model system, 2A5NP presented a complete reversal, where Co@100 was overall the more active species. This brings to light several interesting points. First, by combining the mechanochemical method and varying subsequent calcination temperatures, different mixtures of cobalt oxides and hydroxides can be achieved, the consequence of which dramatically alters both activity and selectivity. Second, it serves caution not to dismiss potential catalysts which underperform with the now ubiquitous 4NP model, and therefore could display enhanced reactivity with other functionalities. Our ongoing efforts focus on understanding the influence of cobalt oxide and hydroxide mixtures which govern the observed reactivity and selectivity with the aim of developing a precise mechanism for NP reduction by early transition-metal oxides.

## Figures and Tables

**Table 1:** Summary of catalytic trials.

Cat.	Substrate.	Change	*k_app_* (min^−1^)	Induction (min)
**--**	4NP	--	no rxn	--
**Co@100**	4NP	--	0.033	2
**Co@350**	4NP	--	0.189	2
**Co@600**	4NP	--	0.057	<2
**Co@100**	4NP	no NaBH_4_	no rxn	--
**Co@350**	4NP	no NaBH_4_	no rxn	--
**Co@600**	4NP	no NaBH_4_	no rxn	--
**Co@350**	4NP	+1 mg KOH	0.132	1
**Co@350**	4NP	+NaBH_4_ last	0.035	30
**Co@350**	4NP	+4NP last	0.032	<5
**KOH**	4NP	--	no rxn	--
**Co_3_O_4_**	4NP	--	no rxn	--
**Co@100**	4A3NP	--	0.004	30
**Co@350**	4A3NP	--	0.114	6
**Co@600**	4A3NP	--	0.003	16
**Co@100**	2A5NP	--	0.047/0.175	8
**Co@350**	2A5NP	--	0.143	28
**Co@600**	2A5NP	--	0.264	22

## Supplementary Materials

The supplementary materials are available online.
