# Supplementary material for: A Combined Mechanochemical and Calcination Route to Mixed Cobalt Oxides for the Selective Catalytic Reduction of Nitrophenols
Source: Molecules. 2019 Dec 25;25(1):89. doi: 10.3390/molecules25010089 (PMC6982874; doi:10.3390/molecules25010089)
Supplement: Supplementary file 1 [file molecules-25-00089-s001.pdf]

Supporting Information

A Combined Mechanochemical and Calcination Route to Mixed Cobalt Oxides for the Selective Catalytic Reduction of Nitrophenols

Lorianne R. Shultz, Bryan McCullough, Wesley J. Newsome, Haider Ali, Thomas E. Shaw, Kristopher O. Davis, Fernando J. Uribe-Romo, Matthieu Baudelet,* Titel Jurca**

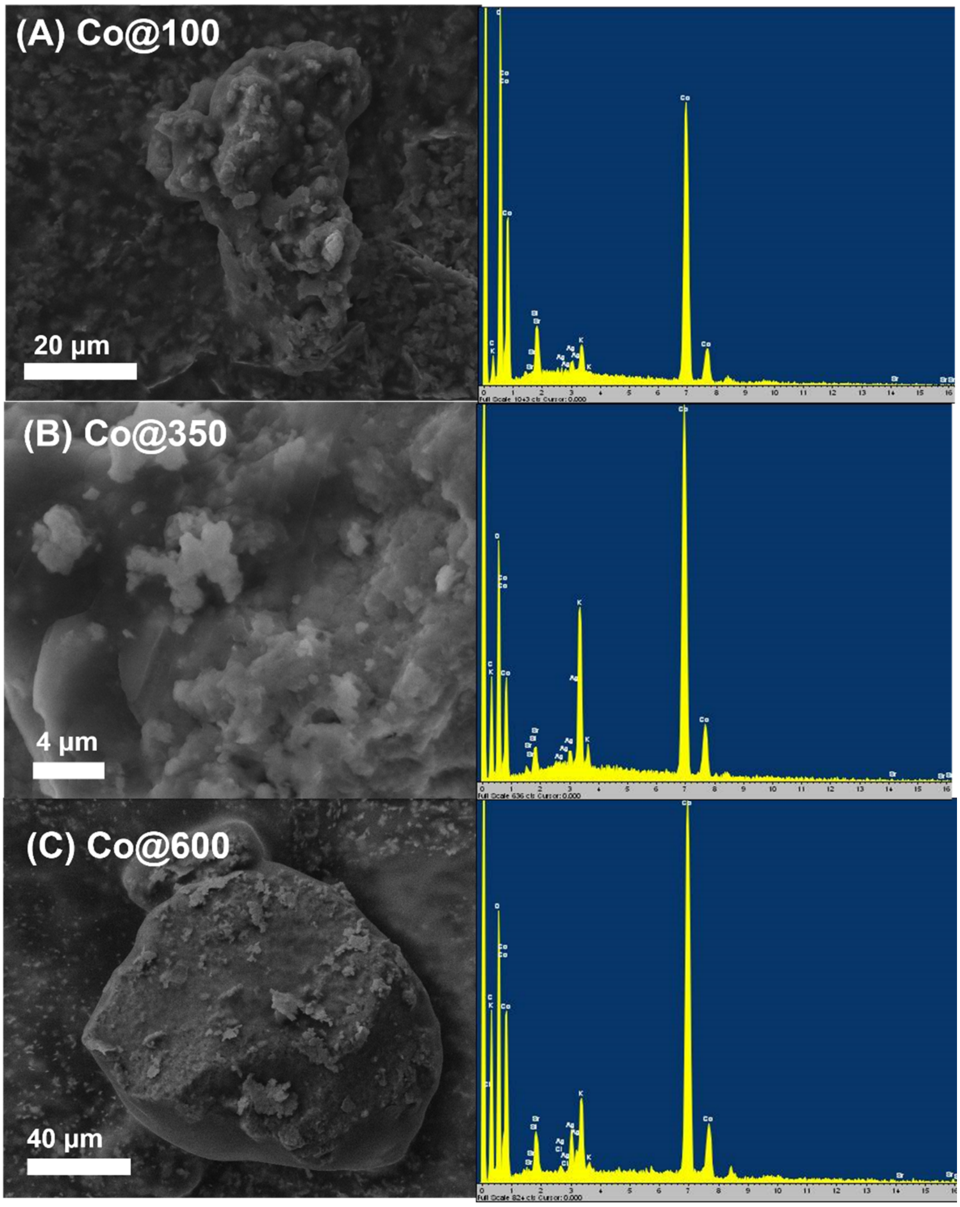

Figure S1: (*left*) Additional representative Scanning Electron Microscopy (SEM) images of (A) **Co@100**, (B) **Co@350**, and (C) **Co@600**; (*right*) representative corresponding SEM-EDX analysis.

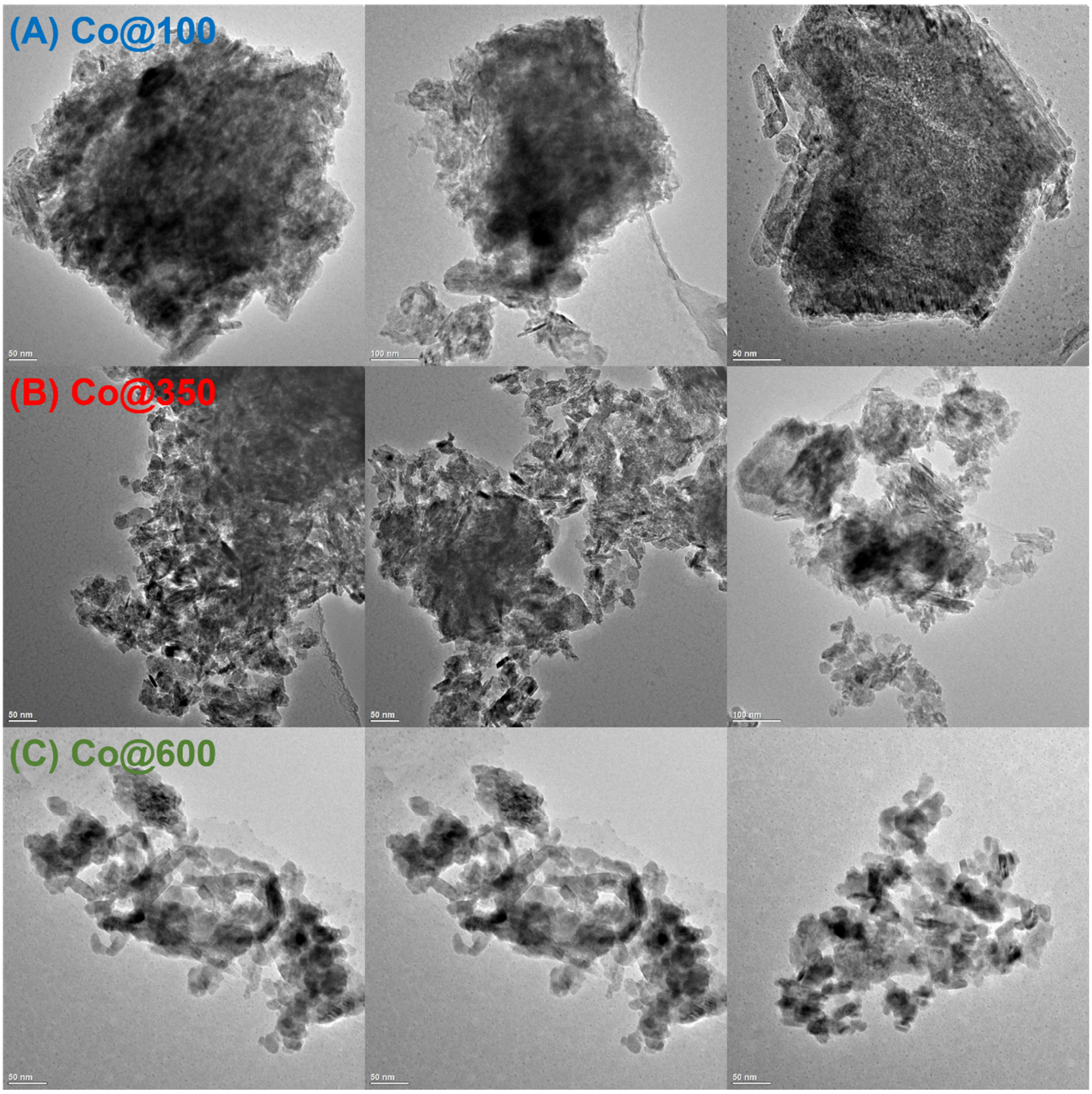

Figure S2: Additional representative Transmission Electron Microscopy (TEM) images of (A) **Co@100**, (B) **Co@350**, and (C) **Co@600**.

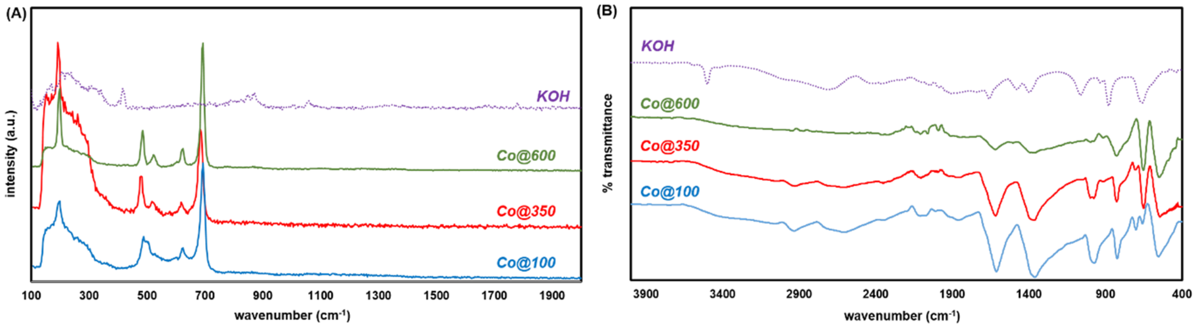

Figure S3: (*left*) Raman and (*right*) FTIR spectra of KOH, **Co@100**, **Co@350** and **Co@600**.

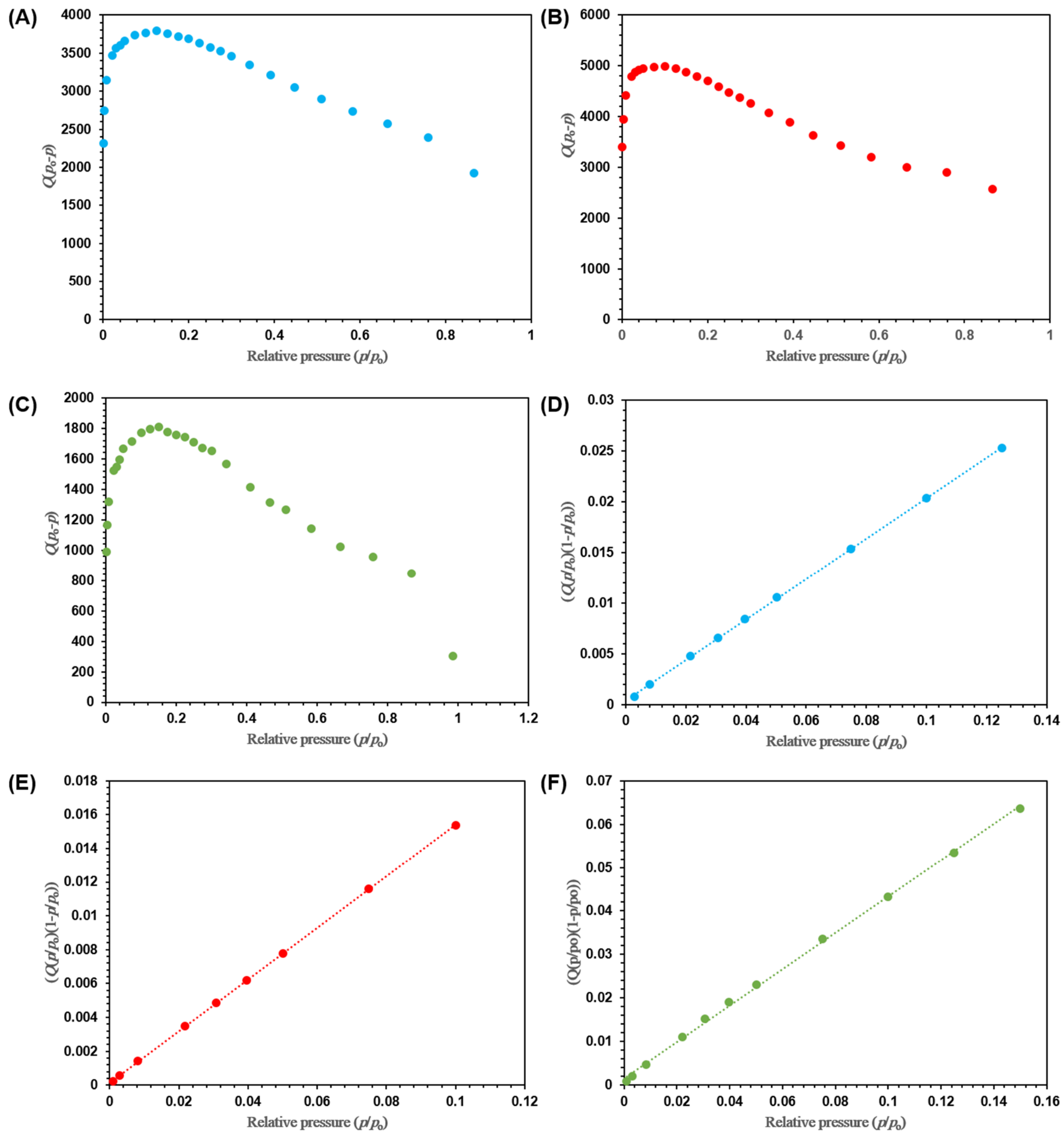

Figure S4: Roquerol plot of (A) **Co@100**, (B) **Co@350**, and (C) **Co@600**; BET plot of (D) **Co@100**, (E) **Co@350**, and (F) **Co@600**.

Table S1. BET parameters, volume of monolayer, surface area and C-parameters of the prepared cobalt oxide particles including the standard deviations.

Sample	Co@100	Co@350	Co@600
**BET slope**	$1.99{\times}10^{-1}$	$1.53{\times}10^{-1}$	$4.18{\times}10^{-1}$
$\sigma_{BET\ slope}$	$8.43{\times}10^{-4}$	$3.46{\times}10^{-4}$	$4.64{\times}10^{-3}$
**BET intercept**	$4.07{\times}10^{-4}$	$1.32{\times}10^{-4}$	$1.57{\times}10^{-3}$
$\sigma_{BET\ intercept}$	$5.39{\times}10^{-5}$	$1.68{\times}10^{-5}$	$3.41{\times}10^{-4}$
V_m (cm^3(STP) g^{-1})	5.00	6.55	2.39
σ_{Vm}	0.02	0.02	0.03
S_{BET} (m^2 g^{-1})	21.75	28.48	10.38
σ_{S_BET}	0.10	0.07	0.12
C_{BET}	491	1121	268
σ_{C_BET}	65	138	58

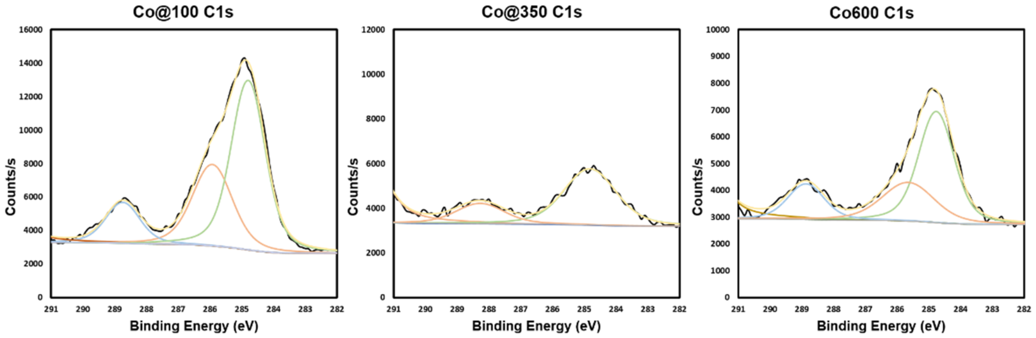

Figure S5: XPS spectra for the C 1s region for **Co@100**, **Co@350** and **Co@600**.

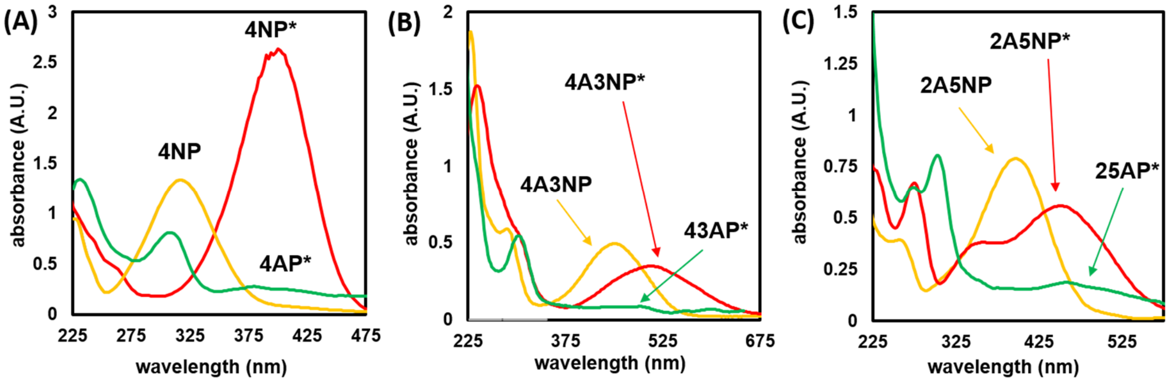

Figure S6: Reference UV-Vis spectra for parent nitrophenol (A-C) (*yellow line*), resulting product from interaction with BH_4^- in solution (*red line*), and respective reduced product/products after catalytic reaction (*green line*).

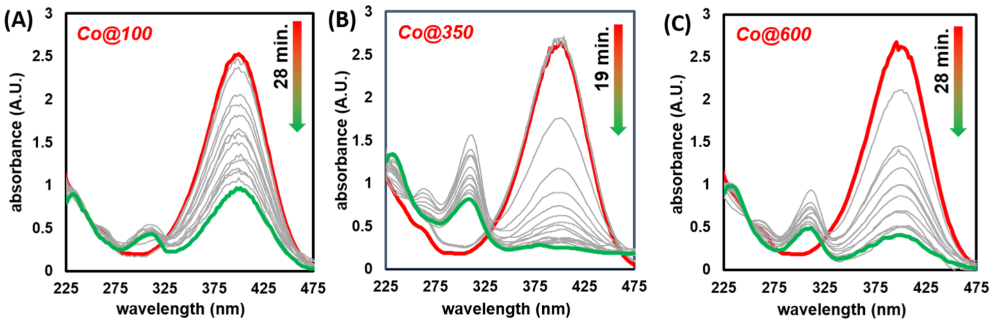

Figure S7: UV-Vis spectra for the reduction of **4NP** by (A) **Co@100**, (B) **Co@350**, and (C) **Co@600**.

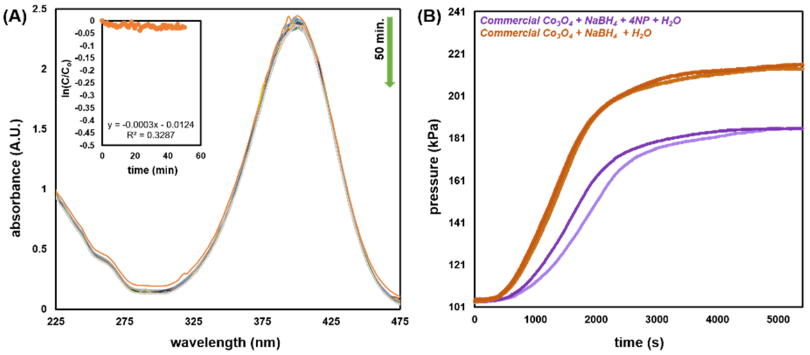

Figure S8: (A) UV-Vis spectrum for the reduction of **4NP** by commercial Co_3O_4 (inset plot of ln(C/Co) as a function of time). (B) Pressure *vs* time for 1 mg Co_3O_4 in 3 mL of DIW; 0.2 mmol of $NaBH_4$, and 1 mg Co_3O_4 in 3 mL of DIW mmol of $NaBH_4$ and 0.39 μmol of **4NP**.

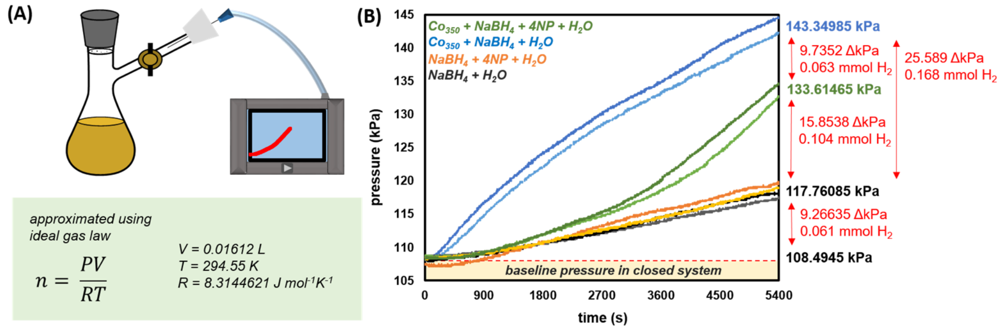

Figure S9: (A) Measurement set-up in a closed system monitored by a Vernier PS400-BTA sensor. (B) A series of measurements resulting from (**grey/black**) 0.2 mmol of $NaBH_4$ in 3 mL of DIW; (**orange/yellow**) 0.2 mmol of $NaBH_4$ and 0.39 μmol of **4NP** in 3 mL of DIW; (**green**) 0.2 mmol of $NaBH_4$, 0.39 μmol of **4NP** and 1 mg **Co@350** in 3 mL of DIW; (**blue**) 0.2 mmol of $NaBH_4$, and 1 mg **Co@350** in 3 mL of DIW. # of mol H_2 evolved approximated with the ideal gas law from the resulting pressure change.

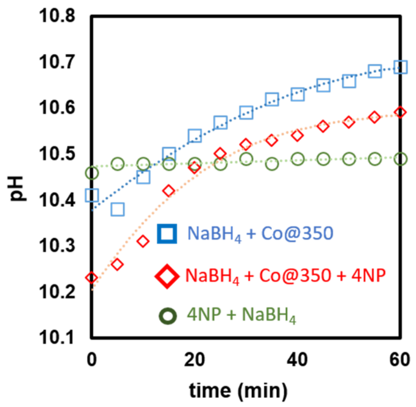

Figure S10: pH measurements for **4NP** + $NaBH_4$; $NaBH_4$ + **Co@350**; **4NP** + $NaBH_4$ + **Co@350**.

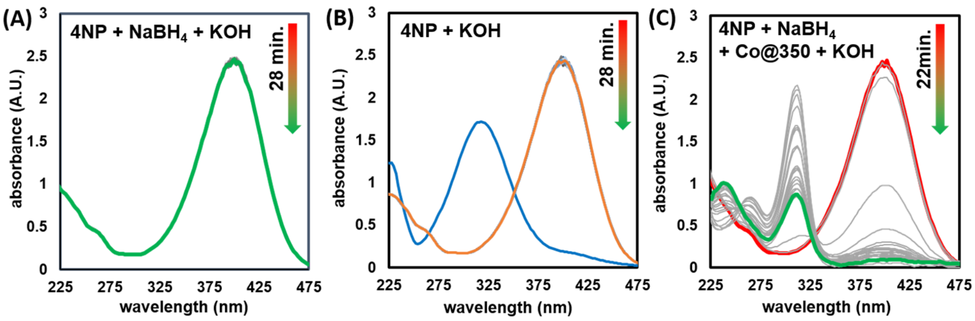

Fig. S11. UV-Vis spectra for the attempted reduction of **4NP** by KOH with (A) and without (B) $NaBH_4$; reduction of **4NP** and $NaBH_4$ with **Co@350** and 1 mg KOH (C).

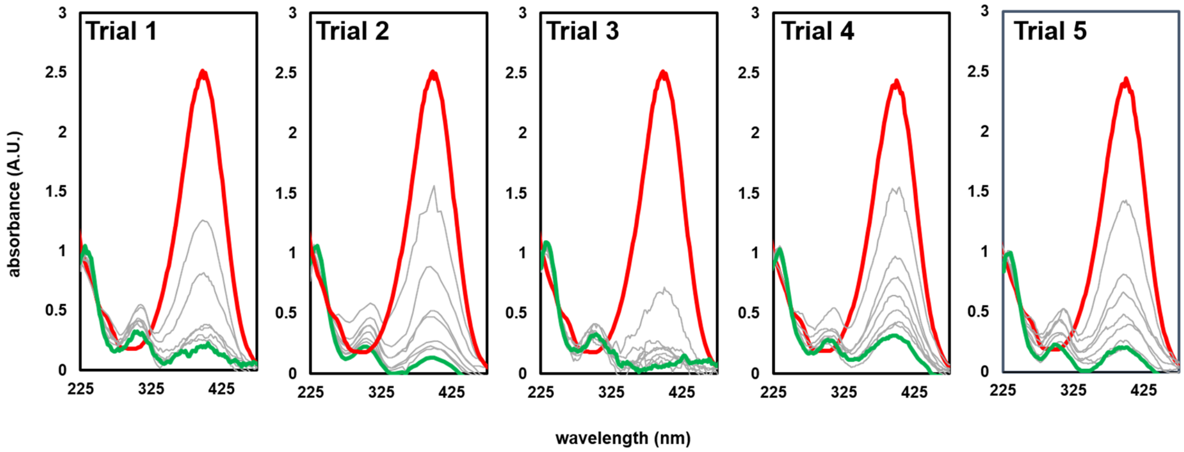

Figure S12: UV-Vis spectra for the reduction of **4NP** by **Co@350** over five successive trials with isolation after each trial.

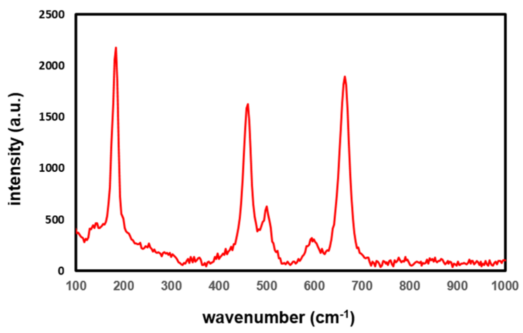

Figure S13: Raman spectra of "spent" **Co@350** after five successive reductions of **4NP** with isolation after each catalytic trial (*i.e. catalyst isolated after completion of Trial 5 in Figure S9*).

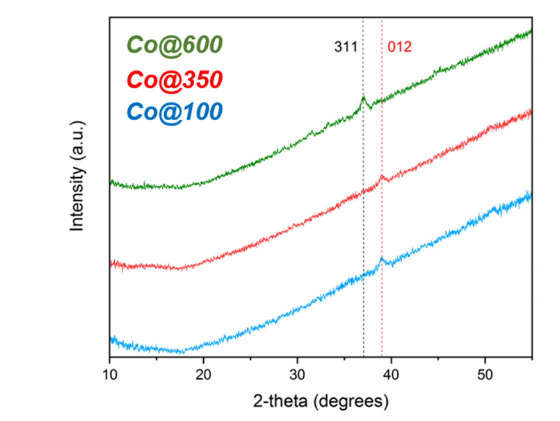

Figure S14: Powder XRD patterns of spent **Co@100**, **Co@350**, and **Co@600**. Indexed by the (311) peak of Co3O4 and (012) peak of CoO(OH).

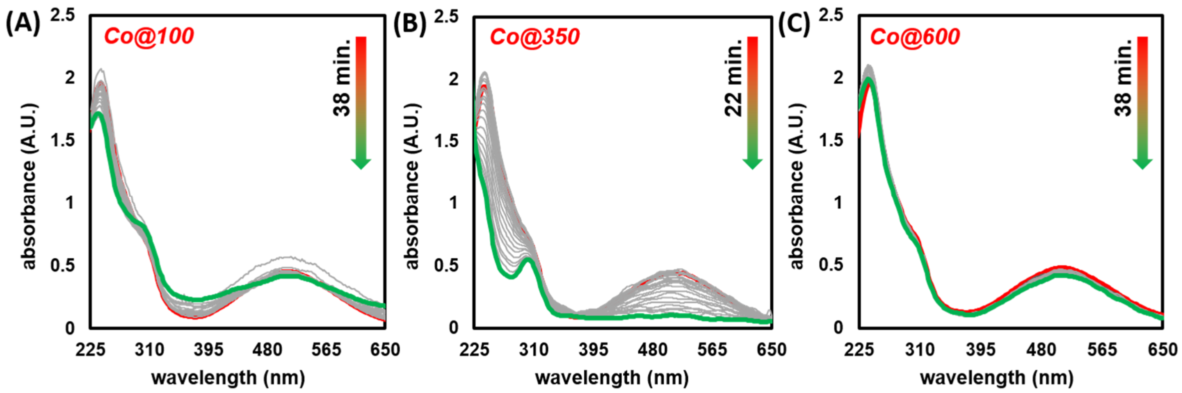

Figure S15: UV-Vis spectra for the reduction of **4A3NP** by (A) **Co@100**, (B) **Co@350**, and (C) **Co@600**.

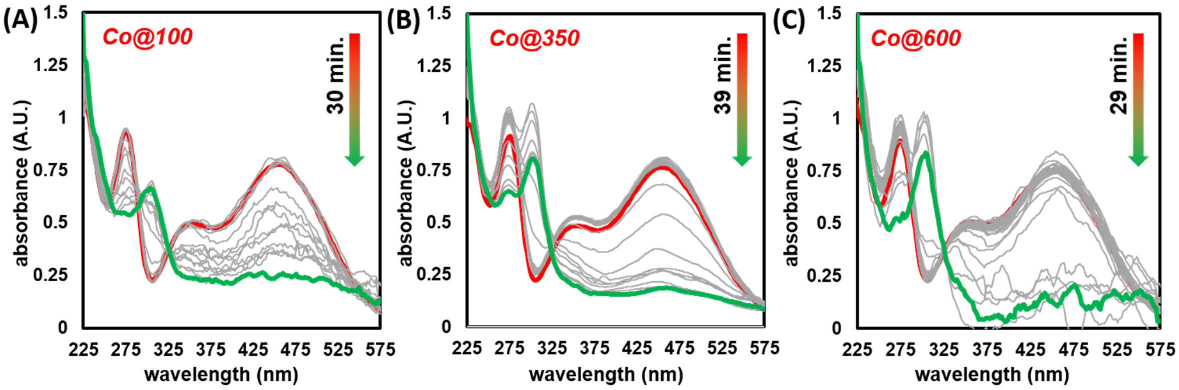

Figure S16: UV-Vis spectra for the reduction of **2A5NP** by (A) **Co@100**, (B) **Co@350**, and (C) **Co@600**.
